# Supplementary material for: Diversity of Monofloral Honey Based on the Antimicrobial and Antioxidant Potential
Source: Antibiotics (Basel). 2022 Apr 28;11(5):595. doi: 10.3390/antibiotics11050595 (PMC9137981; doi:10.3390/antibiotics11050595)
Supplement: Supplementary file 1 [file antibiotics-11-00595-s001.zip › antibiotics-1701472-supplementary.pdf]

BGR/MGR (%) values of honey samples on clinical isolates

	E.coli (2023)	*E. coli* (2067)	*E. coli* (21117)	*M. pachydermatis*	*M. pachydermatis*	*B. cepacian* (2195)	B. cepacian (21952)	*P. vulgaris* (20624)	*P. vulgaris* (20124)	*P. vulgaris* (2147)	*P. aeruginosa* (19117)	*P. aeruginosa* (191211)	*P. aeruginosa* (20129)	*P. aeruginosa* (20722)	*P. aeruginosa* (21615).
MH 10%	84.83	90.72	88.66	118.20	119.66	134.36	134.73	132.91	82.92	89.17	102.32	97.76	95.09	99.73	99.56
MH 15%	58.45	75.52	62.08	62.04	80.51	102.78	110.19	125.69	55.20	77.56	84.68	96.49	91.81	92.66	96.39
MH 20%	25.09	50.82	45.37	28.86	59.41	84.19	82.74	125.94	29.63	66.33	73.03	91.33	73.98	74.33	69.51
BH 10%	103.91	101.86	112.16	81.42	89.24	124.57	131.75	134.72	59.46	89.91	108.81	112.70	111.33	109.48	117.22
BH 15%	75.54	85.54	88.48	58.83	80.39	113.91	129.65	122.92	40.08	67.94	70.78	99.96	104.60	69.11	87.12
BH 20%	54.20	75.52	68.54	57.25	69.67	89.98	108.02	91.74	44.78	60.10	48.85	62.01	58.54	41.42	74.00
AH 10%	87.99	85.22	98.91	58.14	83.89	128.87	121.44	130.52	121.11	128.96	82.71	112.95	110.26	106.21	108.60
AH 15%	81.12	75.51	91.69	51.22	63.52	101.71	116.17	119.49	71.24	81.51	79.49	98.88	90.47	81.81	87.30
AH 20%	45.24	48.48	76.34	47.04	57.60	83.53	84.93	99.16	61.86	66.44	31.14	93.64	87.82	45.98	63.59
LH 10%	97.63	97.59	97.80	65.11	80.39	128.41	134.76	114.43	70.23	89.17	141.09	122.05	113.70	137.48	134.26
LH 15%	63.56	69.85	78.77	53.52	52.66	115.79	130.42	92.96	65.38	75.42	102.99	98.79	98.29	104.24	123.55
LH 20%	48.93	52.35	60.88	52.02	63.14	92.93	90.88	94.21	45.82	54.40	39.62	87.01	81.78	61.80	77.36
C	100.02	99.98	99.98	100.04	100.02	100.00	100.02	100.40	100.00	99.98	100.05	100.04	100.04	100.02	100.05

BGR/MGR (%) values of honey samples on ATCC strains

The concentrati of honey %	*S. pyogenes* (ATCC 19615)	*S. aureus* (ATCC 25923)	*S. flexneri* (ATCC 12022)	*P. aeruginosa* (ATCC 27853)	*E. coli* (ATCC 25922)	*S. typhimurium* (ATCC 14028)	*H. influenzae* (ATCC 10211)	*C. parapsilopsis* (ATCC 22019)	*C. albicanson* (ATCC 10231)
MH 10	79.99	88.18	96.05	100.10	96.04	78.14	53.93	88.66	92.15
MH 15	77.10	80.56	93.86	118.31	74.94	86.10	65.13	93.95	86.26
MH 20	67.73	77.37	90.01	120.46	66.16	86.29	70.22	100.97	72.91
BH 10	91.53	98.67	95.01	98.81	74.58	86.77	78.03	99.57	81.35
BH 15	94.89	99.73	89.35	86.07	83.05	88.30	85.15	120.19	94.80
BH 20	97.40	104.85	76.83	77.98	94.17	93.67	91.70	131.32	98.72
AH 10	103.92	98.03	104.68	118.94	98.58	117.16	91.00	140.93	75.86
AH 15	98.98	108.89	123.73	97.08	73.74	108.72	94.77	130.24	80.47
AH 20	88.97	112.73	125.98	90.75	68.87	88.69	94.91	119.98	88.62
LH 10	112.52	116.24	91.47	118.81	86.35	136.82	73.83	114.33	111.25
LH 15	102.55	123.80	86.19	119.60	93.18	116.75	78.94	121.53	106.97
LH 20	96.64	126.78	82.34	120.80	94.37	100.58	84.91	127.75	102.00
C	100.00	100.05	100.00	100.03	100.03	100.00	100.00	99.96	100.00
